# Accuracy of ultrasound in predicting thyroid malignancy: a comparative analysis of the ACR TI-RADS and ATA risk stratification systems

**DOI:** 10.20945/2359-4292-2023-0245

**Published:** 2024-04-26

**Authors:** Shaza Samargandy, Aliaa H. Ghoneim

**Affiliations:** 1 King Abdulaziz University Department of Medicine Endocrine Unit Jeddah Saudi Arabia Endocrine Unit, Department of Medicine, King Abdulaziz University, Jeddah, Saudi Arabia; 2 King Abdulaziz University Radiology Department Jeddah Saudi Arabia Radiology Department, King Abdulaziz University, Jeddah, Saudi Arabia

**Keywords:** Thyroid nodule, thyroid cancer, diagnostic ultrasound, fine-needle aspiration, risk assessment, risk factors

## Abstract

**Objective:**

Thyroid nodules are very common in clinical practice, and ultrasound has long been used as a screening tool for their evaluation. Several risk assessment systems based on ultrasonography have been developed to stratify the risk of malignancy and determine the need for fine-needle aspiration in thyroid nodules, including the American Thyroid Association (ATA) system and the American College of Radiology Thyroid Imaging Reporting and Data System (ACR TI-RADS). The aim of this study was to compare the performance of the ATA and ACR TI-RADS systems in predicting malignancy in thyroid nodules based on the nodules' final histopathology reports.

**Materials and methods:**

We performed a retrospective review of medical records to identify patients who underwent thyroid surgery at King Abdulaziz University from 2017 to 2022. The ultrasound features of the nodules with confirmed histopathology (benign versus malignant) were evaluated. Both ATA and ACR TI-RADS scores were documented.

**Results:**

The analysis included 191 patients who underwent thyroid surgery and fulfilled the inclusion criteria. Hemithyroidectomy was performed in 22.5% of the patients, and total thyroidectomy was performed in 77.0% of them. In all, 91 patients (47.6%) were found to have malignant nodules on histopathology. We then compared the histopathology reports with the preoperative ultrasonographic risk scores. The estimated sensitivity and specificity in identifying malignant nodules were, respectively, 52% and 80% with the ATA system and 51.6% and 90% with the ACR TI-RADS system.

**Conclusion:**

Both ATA and ACR TI-RADS risk stratification systems are valuable tools for assessing the malignancy risk in thyroid nodules. In our study, the ACR TI-RADS system had superior specificity compared with the ATA system in predicting malignancy among high-risk lesions.

## INTRODUCTION

Thyroid nodules are common clinical findings that affect nearly 50% of the population worldwide (1). While the majority of these nodules are benign, it is crucial to identify those that are malignant, especially in countries with a high prevalence of thyroid cancer (2). In Saudi Arabia, where the present study was conducted, thyroid cancer is the second most common malignancy in women (3).

Ultrasound has been a valuable tool for evaluating thyroid nodules for many years. Ultrasonographic features, along with other clinical features like history of head and neck radiation and familial thyroid cancer, can guide clinical decisions regarding fine-needle aspiration (FNA) biopsy of concerning nodules or be used for surveillance (4). To assess the risk of malignancy in these nodules, several risk stratification systems based on ultrasonography have been developed, including the American Thyroid Association (ATA) risk stratification system and the American College of Radiology Thyroid Imaging Reporting and Data System (ACR TI-RADS). These scoring systems examine the ultrasonographic features of a thyroid nodule associated with an increased risk of malignancy, which include a solid composition, hypoechogenicity, calcifications, and a taller-than-wide shape. Based on the ultrasonographic features, the nodules are assigned to risk categories (5,6).

Studies concerning the diagnostic performance of the 2015 ATA and 2017 ACR TI-RADS risk stratification systems have yielded variable results. A study from China performing a head-to-head comparison of both systems found a better accuracy for the 2015 ATA (92.1%) than the ACR TI-RADS (89.25%) system, with almost similar areas under the curve (0.92 and 0.93, respectively) (7).

Based on these considerations, the aim of the present study was to compare the performance of the ATA *versus* ACR TI-RADS risk stratification systems in predicting the occurrence of malignancy in thyroid nodules. To the best of our knowledge, no direct comparison between the two risk stratification systems has been conducted in the Saudi population, especially using postoperative histopathology, which is the gold standard for the diagnosis of malignancy.

## MATERIALS AND METHODS

The present study was approved by the Unit of Biomedical Ethics at King Abdulaziz University.

### Data collection

**Figure 1:**
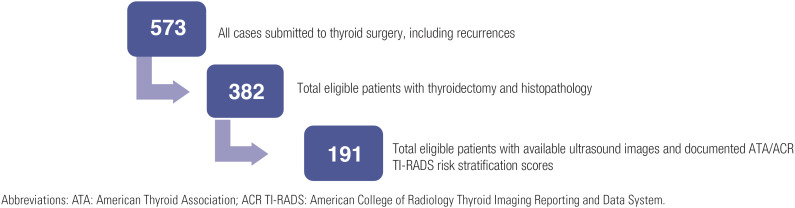
Flow diagram of patient's inclusion.

We conducted a retrospective review of all patients who underwent thyroid surgery from 2017 to 2022 at King Abdulaziz University. The final pathology result (if benign or malignant) was documented for each patient. The malignant cases included differentiated, medullary, anaplastic, and poorly differentiated thyroid carcinomas. We evaluated the ultrasonographic features of all thyroid nodules and recorded their reported ATA and ACR TI-RADS risk scores. We included only cases in which both ATA and ACR TI-RADS scores were documented by the reporting radiologist or with detailed reporting of the ultrasonographic features to enable the assignment of the risk score for each nodule. Figure 1 shows a flow diagram of the patients’ inclusion in the study.

The ultrasound evaluations were performed by a radiologic technologist using a high-frequency linear transducer (10 MHz) and were reviewed by a consultant radiologist specialized in thyroid imaging, who confirmed the final risk stratification score. The images were also evaluated by a head and neck radiologist and a consultant endocrinologist for score confirmation. Data regarding the patients’ demographics, preoperative TSH level, and preoperative FNA results were collected when available. Pediatric patients were excluded from the study.

According to the ACR TI-RADS risk stratification system, the nodules were categorized as TR1 (benign), TR2 (not suspicious), TR3 (mildly suspicious), TR4 (moderately suspicious), and TR5 (highly suspicious). As for the ATA score, the nodules were categorized as benign (cysts), very low, low, intermediate, and high risk. Results from FNA were reported according to the Bethesda System for Reporting Thyroid Cytopathology, and the categories included nondiagnostic (Bethesda I), benign (Bethesda II), atypia of undetermined significance/follicular lesion of undetermined significance (AUS/FLUS; Bethesda III), follicular neoplasm (Bethesda IV), suspicious for malignancy (Bethesda V), and malignant (Bethesda VI) (8).

### Statistical analysis

To facilitate a direct comparison between the two scoring systems, we categorized the ultrasonographic risk as "positive US" (highest chance of malignancy) or "negative US." The TR1, TR2, TR3, and TR4 scores were categorized as "negative US," while TR5 was categorized as "positive US." As for the ATA risk stratification system, the nodules with benign (cysts), very low, low, and intermediate suspicion were categorized as "negative US," while those with high suspicion were categorized as "positive US." The nodules grouped as "positive US" and "negative US" were then linked to the nodules’ final histopathology. Finally, we calculated the sensitivity, specificity, positive predictive (PPV), and negative predictive (NPV) values of the highest scores in each risk stratification system.

All statistical analyses were performed using the software SPSS, version 18.0 (IBM Corp., Armonk, NY, USA).

## RESULTS

A total of 191 patients fulfilled the inclusion criteria, all of whom had undergone surgery due to a non-benign result on FNA or to relieve compressive symptoms from a benign goiter. Hemithyroidectomy was performed in 22.5% of the patients, while total thyroidectomy was performed in 77.0% of them. The patients in the study sample had a mean age of 43.4 years (interquartile range [IQR] 14-80 years), and most of them (83.3%) were female. On ultrasound, the nodules had a mean size of 3.0 cm (IQR 1.8-4 cm) and a maximum size of 9 cm. The mean preoperative TSH level was 2.55 ± 5.6 µIU/mL. After surgery, 91 patients (47.6%) had confirmed malignancy on thyroid histopathology.

Each ultrasound image was assigned an ACR TI-RADS score and an ATA score. In all, 68 nodules were categorized as "high suspicion" according to the ATA system; of these, 50 were categorized as TR5 and 18 as TR4. Table 1 illustrates the relationship between the ATA and ACR TI-RADS scores in the study sample.

**Table 1:** Relationship between American Thyroid Association (ATA) and American College of Radiology Thyroid Imaging Reporting and Data System (ACR TI-RADS) risk stratification scores in the study sample

	ATA					
	Benign (cysts)	Very low risk	Low risk	Intermediate risk	High risk	Total*
TR1	10	5	10	0	0	25 (13.1%)
TR2	1	4	13	1	0	19 (9.9%)
TR3	0	1	31	9	0	41 (21.5%)
TR4	0	0	11	20	18	49 (25.7%)
TR5	0	0	4	3	50	57 (29.8%)
Total**	11	10	69	33	68	191 (100.0%)

The data are shown as number of nodules or number (percentage) of nodules.

* Total number (percentage) of nodules within each ACR TI-RADS category.

** Total number of nodules within each ATA category.

ATA: risk category according to the American Thyroid Association system; TR: risk category according to the American College of Radiology Thyroid Imaging Reporting and Data System.

A total of 187 FNA biopsies were performed, as four patients declined to undergo this procedure. Table 2 shows the numbers and percentages of nodules divided according to final histopathology results (benign *versus* malignant) with their corresponding preoperative ATA and ACR TI-RADS risk scores.

**Table 2:** Number (percentage) of nodules divided according to final histopathology results (benign *versus* malignant) with their corresponding preoperative ATA and ACR TI-RADS risk score (cancer prediction)

	Histopathology		Total
	Benign	Malignant	
Benign (ATA)	10 (90.9%)	1 (9.1%)	11 (100.0%)
TR1 (ACR TI-RADS)	19 (76.0%)	6 (24.0%)	25 (100%)
Very low risk (ATA)	6 (60.0%)	4 (40.0%)	10 (100.0%)
TR2 (ACR TI-RADS)	15 (8.9%)	4 (21.1%)	19 (100%)
Low risk (ATA)	45 (65.2%)	24 (34.8%)	69 (100.0%)
TR3 (ACR TI-RADS)	27 (5.9%)	14 (34.1%)	41 (100%)
Intermediate risk (ATA)	19 (57.6%)	14 (42.4%)	33 (100.0%)
TR4 (ACR TI-RADS)	29 (8.0%)	21 (42.0%)	50 (100%)
High risk (ATA)	20 (29.4%)	48 (70.6%)	68 (100.0%)
TR5 (ACR TI-RADS)	10 (7.5%)	47 (82.5%)	57 (100%)

ATA: risk category according to the American Thyroid Association; TR: risk category according to the American College of Radiology Thyroid Imaging Reporting and Data System. The data are provided as number (percentage) of nodules within each risk score.

### Performance of the ATA risk stratification system

Among nodules with high suspicion according to the ATA system, the most common FNA result (25% of the nodules) was Bethesda III (AUS/FLUS). On the other hand, malignant FNA results were found in 20.6% of the nodules with high suspicion according to the ATA system. Supplementary Table 1 presents the complete results of the analysis between ATA risk scores and corresponding FNA results according to the Bethesda System.

A total of 70.6%, 42.4%, and 34.8% of the nodules with, respectively, high, intermediate, and low risk suspicion according to the ATA system were found to have thyroid cancer on histopathology. The detailed findings of this analysis are shown in Supplementary Table 1, and the relationship between ATA risk scores and malignancy is shown in Table 2.

The categorization of the nodules according to ultrasonographic risk yielded 68 nodules in the "positive US" group based on the ATA risk score. This scoring system had the following performance in accurately predicting malignancy in "positive US" nodules: 52% sensitivity, 80.0% specificity, 70% PPV, and 65% NPV (Table 3).

**Table 3:** Performance of the American Thyroid Association risk stratification in accurately predicting malignancy

ATA risk score	Final histopathology result		
	Benign	Malignant	Total
Negative US	80	43	123
Positive US	20	48	68
Total	100	91	191

The data are shown as number of nodules. Abbreviations – ATA: American Thyroid Association; negative US:nodules with benign (cysts), very low, low, and intermediate suspicion according to the ATA system; positive US: nodules with high suspicion according to the ATA system.

### Performance of the ACR TI-RADS risk stratification system

Among nodules with a TR5 score according to the ACR TI-RADS system, the most common FNA result (24.6% of the nodules) was Bethesda III (AUS/FLUS). On the other hand, malignant FNA results were found in 24.6% of the nodules categorized as TR5. Supplementary Table 2 presents the complete analysis of the ACR TI-RADS risk scores and the corresponding FNA results according to the Bethesda System.

A total of 82.5%, 42.0%, and 34.1% of the nodules categorized as, respectively, TR5, TR4, and TR3 were found to be malignant on histopathology. Table 2 shows the relationship between the ACR TI-RADS risk scores and malignancy.

The categorization of the nodules according to ultrasonographic risk yielded 57 nodules in the "positive US" group based on the ACR TI-RADS risk score. This scoring system had the following performance in accurately predicting malignancy in "positive US" nodules: 51.6% sensitivity, 90% specificity, 82.5% PPV, and 67.2% NPV (Table 4).

**Table 4:** Performance of the American College of Radiology Thyroid Imaging Reporting and Data System risk stratification in accurately predicting malignancy

ACR TI-RAD risk score	Final histopathology result		
	Benign	Malignant	Total
Negative US	90	44	134
Positive US	10	47	57
Total	100	91	191

The data are shown as number of nodules. Abbreviations – ACR TI-RADS: American College of Radiology Thyroid Imaging Reporting and Data System; negative US: nodules categorized as TR1, TR2, TR3, and TR4 according to the ACR TI-RADS system; positive US: nodules categorized as TR5 according to the ACR TI-RADS system.

We further compared the accuracy of the two risk stratification systems. The calculated overall accuracy rates were 67.0% for the ATA system and 71.7% for the ACR TI-RADS system. There was no significant difference in accuracy rates between the two systems (p = 0.27).

## DISCUSSION

The results of the present study showed that the ACR TI-RADS compared with the ATA risk stratification system had a higher specificity in predicting malignancy among high-risk lesions, *i.e.*, those categorized as TR5 (ACR TI-RADS) and as "high suspicion" (ATA). However, both systems had comparable performances regarding nodules with all other risk scores in our sample.

To the best of our knowledge, this is the first head-to-head comparison of the ATA and ACR TI-RADS systems in Saudi Arabia based on final histopathology results. Several studies published elsewhere have compared these two scoring systems, but in most of these studies, the diagnosis of benign lesions was based solely on FNA. A study from China published by Wu and cols. using thyroid FNA as the diagnostic standard also showed a higher specificity (66%) and accuracy for the ACR TI-RADS compared with the ATA scoring system, but a higher sensitivity with the ATA system (93.4%) (9). A meta-analysis by Joo and cols. showed results comparable to ours, as the pooled sensitivity between the two systems was very close in their study (89% for the ATA and 82% for the ACR TI-RADS). These authors also found a higher specificity with the ACR TI-RADS compared with the ATA system (60% *versus* 34%, respectively) (10).

Since ultrasound is an operator-dependent method, and the prevalence of thyroid cancer differs from country to country, variations between studies regarding sensitivity, specificity, and risk of malignancy are expected. In the 2015 ATA guidelines for thyroid nodules and thyroid cancer, the estimated risk of malignancy among nodules with high suspicion is 70%-90%, and in the 2017 ACR TI-RADS guidelines, the estimated risk of malignancy in TR5 lesions is >20% (11). However, in a systematic review and meta-analysis of eight studies by Kim and cols., the pooled risk of malignancy was 55.4%, 34.2%, and 12.2% among nodules with, respectively, high, intermediate, and low suspicion according to the ATA system, and 59.3%, 20.7%, and 11.0% among nodules categorized as, respectively, TR5, TR4, and TR3 scores according to the ACR TI-RADS system (12). In our sample, the risk of malignancy was higher across risk categories in both systems. With the ATA system, the risk of malignancy was 70.6%, 42.4%, and 34.8% for high, intermediate, and low risk lesions, respectively, while for the ACR TI-RADS system, the risk of malignancy was 82.5%, 42.0%, and 34.1% for TR5, TR4, and TR3 lesions, respectively.

Studies from Saudi Arabia examining the correlation between ACR TI-RADS risk scores and risk of malignancy have shown that 86.5% of the nodules categorized as TR5 are malignant; this percentage is comparable to the one found in the present study (82.5%) (13). Another Saudi study using FNA as the standard for determining the malignant potential associated with each ACR TI-RADS category found a risk of malignancy of 0% for TR1 and TR2, 3.7% for TR3, 6.6% for TR4, and 22.7% for TR5 lesions (14). In our sample, the diagnostic standard was the final histopathology. Had we adopted cytopathologic examination, our results would not differ much. Indeed, when we analyzed only nodules categorized as Bethesda VI, we found no cases of Bethesda VI among TR1 lesions, but this Bethesda category was present in 5.6%, 7.9%, 6.1%, and 24.5% of the TR2, TR3, TR4, and TR5 lesions, respectively.

The present study has some strengths and limitations. As strengths, the study was unique in being the first to compare the ATA and ACR TI-RADS systems using real-world data from Saudi Arabia. Also, the study used the histopathology report, which is the gold standard method to identify malignant and benign lesions. The limitations of the study include the retrospective, single-center design and the fact that it was conducted at a tertiary-care academic center; therefore, the results may not apply to other centers. Additionally, we did not exclude incidental microcarcinomas when we labeled the case as malignant on final pathology. We also acknowledge that we only included cases with ATA and ACR TI-RADS scores mentioned in the report or at least with sufficient data to identify the scores accurately. Finally, the assessment was not done individually, and not all radiologists had expertise in thyroid ultrasound.

In conclusion, both ATA and ACR TI-RADS risk stratification systems are useful tools in managing thyroid nodules and have comparable sensitivity, but the ACR TI-RADS had better specificity and PPV. Knowledge about the accuracy and limitations of each of these systems and the incorporation of results from cytologic examination can help direct the management of patients with thyroid nodules.

## Supplementary Tables

**Supplementary Table 1:** American Thyroid Association risk scores and corresponding fine-needle aspiration results according to the Bethesda System for Reporting Thyroid Cytopathology

ATA risk score	FNA result						Total
	Bethesda I	Bethesda II	Bethesda III	Bethesda IV	Bethesda V	Bethesda VI	
Benign	5 (45.5%)	3 (27.3%)	3 (27.3%)	0 (0.0%)	0 (0.0%)	0 (0.0%)	11 (100.0%)
Very low risk	1 (12.5%)	4 (50.0%)	2 (25.0%)	0 (0.0%)	0 (0.0%)	1 (12.5%)	8 (100.0%)
Low risk	8 (12.1%)	27 (40.9%)	18 (27.3%)	7 (10.6%)	3 (4.5%)	3 (4.5%)	66 (100.0%)
Intermediate risk	1 (3.1%)	12 (37.5%)	7 (21.9%)	5 (15.6%)	5 (15.6%)	2 (6.2%)	32 (100.0%)
High risk	6 (9.2%)	8 (12.3%)	17 (26.2%)	9 (13.8%)	11 (16.9%)	14 (21.5%)	65 (100.0%)
Total	21 (11.5%)	54 (29.7%)	47 (25.8%)	21 (11.5%)	19 (10.4%)	20 (11.0%)	182 (100.0%)

The data are shown as number (percentage) of nodules. Abbreviations – ATA: American Thyroid Association; FNA: fine-needle aspiration.

**Supplementary Table 2:** American College of Radiology Thyroid Imaging Reporting and Data System risk scores and corresponding fine-needle aspiration results according to the Bethesda System for Reporting Thyroid Cytopathology

ACR TI-RADS risk score	FNA result						Total
	Bethesda I	Bethesda II	Bethesda III	Bethesda IV	Bethesda V	Bethesda VI	
TR1	7 (29.2%)	7 (29.2%)	8 (33.3%)	2 (8.3%)	0 (0.0%)	0 (0.0%)	24 (100.0%)
TR2	3 (16.7%)	10 (55.6%)	2 (11.1%)	1 (5.6%)	1 (5.6%)	1 (5.6%)	18 (100.0%)
TR3	1 (2.6%)	19 (50.0%)	10 (26.3%)	4 (10.5%)	1 (2.6%)	3 (7.9%)	38 (100.0%)
TR4	6 (12.2%)	16 (32.7%)	13 (26.5%)	6 (12.2%)	5 (10.2%)	3 (6.1%)	49 (100.0%)
TR5	4 (7.5%)	2 (3.8%)	14 (26.4%)	8 (15.1%)	12 (22.6%)	13 (24.5%)	53 (100.0%)
Total	21 (11.5%)	54 (29.7%)	47 (25.8%)	21 (11.5%)	19 (10.4%)	20 (11.0%)	182 (100.0%)

The data are shown as number (percentage) of nodules. Abbreviations – ACR TI-RADS: American College of Radiology Thyroid Imaging Reporting and Data System; FNA: fine-needle aspiration.
